# Smart energy management in residential buildings: the impact of knowledge and behavior

**DOI:** 10.1038/s41598-024-51638-y

**Published:** 2024-01-19

**Authors:** Baraa Hakawati, Allam Mousa, Fadi Draidi

**Affiliations:** 1 An-Najah National University, Nablus, Palestine; 2 AI & VR Research Center, An-Najah National University, Nablus, Palestine

**Keywords:** Environmental sciences, Environmental impact

## Abstract

A new technology called smart energy management makes use of IoT concepts to enhance energy efficiency and lower waste in structures. The goal of this study is to comprehend how household energy management knowledge affects energy usage, user behavior, related expenses, and environmental effect. Through a survey of 100 valid replies in Palestine, the research model assessed the knowledge and consumption habits of building occupants. Smart PLS software was used to analyze the research model using partial least squares structural equation modeling (PLS-SEM). Using path coefficients and behavior as a mediating variable, the structural model connected the latent variables. The mediation hypotheses were tested using the Preacher and Hayes method, and the indirect effect and confidence intervals were estimated and calculated using bootstrapping. The findings demonstrated that by lowering energy use and enhancing overall building performance, residential buildings that implement smart energy consumption management systems may move toward a more sustainable future. Furthermore, the study found that education and awareness campaigns are necessary to increase residents’ knowledge of these systems to promote energy savings. The results also indicated statistically significant indirect effects, supporting the existence of mediation of the behavior construct. Path coefficient values and *P*-values were presented to further support the study’s hypotheses. Such smart energy management systems represent an important innovation in building management and can help create more sustainable and efficient buildings.

## Introduction

The sudden rise in industrial, economic and population growth worldwide has led to a serious escalation in energy consumption, which has led to the need to increase energy generation efforts to counter this increase. Unfortunately, this increase in power generation carries the double burden of prohibitive costs and negative effects on air quality. Moreover, it opens the door to potential environmental and political challenges associated with resource depletion, global warming, and acid rain. Addressing these challenges necessitates a more rational and efficient management of energy consumption through contemporary methods that optimize energy utilization and mitigate wastage.

The contemporary landscape is marked by the ascendancy of smart techniques and technologies, with the Internet of Things (IoT) being a notable exemplar^1^. Building upon this^2^, introduced a thought-provoking methodology, a public opinion survey that delineates the technologies and advantages underpinning IoT’s implementation in the factory sector. Our study, focused on the residential West Bank market in Palestine, seeks to extend this understanding, though without the inclusion of smart electric meters, as expounded by^2^.

An exploration of the energy landscape in Palestine reveals a significant contribution of the construction and buildings sector, accounting for approximately 57% of the total energy consumption between 2011 and 2019, dwarfing the industrial sector’s share, which stands at a mere 10%^3^. This context frames our study’s purpose, aiming to assess the readiness of the residential buildings sector in the West Bank to embrace smart systems for energy management. Central to this endeavor is research into effective applications for smart energy consumption management within residential buildings, with a focus on reducing energy waste and enhancing energy efficiency. Residents’ behavior and knowledge of smart management are central to this exploration, and their insights will be obtained through a pool of residents’ opinions. The study also aims to ascertain the level of awareness among the population regarding these systems, and their ability to reduce environmental damage and compensate for the increasing costs of energy consumption.

Contemporary global concern about the wise use of energy is driven by the growing mandate for energy and the escalating environmental impacts of energy consumption. This necessity is most acutely demonstrated in Palestine, where the inhabited sector accounts for a large portion of the country's energy consumption. Thus, the quest to delve into the intelligent management of indoor energy consumption is more urgent than ever. While cost-related aspects have typically dominated discussions about smart energy consumption management, our study seeks to provide a comprehensive understanding that includes knowledge of residents, residential behavior, and environmental implications.

The necessity of this study arises from the rapid rise in global industrial, economic and population growth, which has led to a significant increase in energy consumption. This escalation underscores the urgent need for a wiser and more effective approach to managing energy consumption through modern methodologies. The current landscape is characterized by the dominance of smart technologies and techniques, with the Internet of Things (IoT) emerging as a prominent model. Against this background, the study seeks to assess the readiness of the residential building sector in the West Bank to adopt and integrate smart energy management systems, with the aim of reducing energy waste and enhancing overall efficiency by studying residents’ knowledge of these technologies along with their consumption behavior inside buildings. The focal point of our research lies in understanding key aspects of residents’ understanding of the intelligent management of energy consumption within buildings, along with their patterns of energy consumption. This endeavor seeks to unravel the complex interplay between these factors, particularly in the unique context of the Palestinian homeland. Furthermore, our research seeks to elucidate the determinants affecting the incorporation of smart energy management protocols into building designs and characterize their effects on energy efficiency and financial dynamics.

The outcomes of this study are prepared to provide invaluable insights to policy makers and stakeholders, enabling the formulation of effective strategies to increase energy efficiency and reduce the environmental burden of energy consumption in the residential sector in Palestine. Through this research, a host of discoveries are expected to emerge and, perhaps most importantly, shed light on the relationship between residents' knowledge and behavior within homes, and their implications for costs and the environment. In addition, an exploration of the prevalence of smart systems in regional homes over recent years, along with insights into the technologies prevailing in the Palestinian construction market, will enrich our understanding.

In order to transition our energy system towards sustainable production and consumption, it is crucial to effectively involve consumers, encouraging them to actively participate in this endeavor. One method to achieve this is through manual demand response, wherein end users react to variations in energy production, thereby assisting in grid stabilization by modifying their consumption patterns^4^.

In the current literature, the global buildings sector, a prominent contributor to energy consumption, relies heavily on electricity for various amenities, as noted by^5^. Zhao and Magoulès^6^ underscore the multitude of factors influencing energy use in buildings, including interior design, external conditions, lighting, air conditioning, and occupant-related activities. The swift advancements in science and technology, as exemplified by the widespread adoption of high-capacity mobile devices^5^, have extended to electrical installations in homes and buildings, encompassing applications such as lighting, heating, ventilation, air conditioning, security, and energy consumption management^7^.

The integration of smart technologies in buildings, facilitated by the Internet of Things (IoT), as highlighted by^8^, plays a pivotal role in enhancing user comfort and overall technological features. Smart homes, equipped with technologies for customized services^8^, are recommended for monitoring energy consumption and improving efficiency through computational intelligence solutions. Various functions, including Energy Use Control (EUC), Energy Performance Analysis (EPA), Energy Consumption Monitoring (ECM), and Energy Demand Forecasting (EDF), contribute to managing energy use^9^.

Building-Energy-Management-Systems (BEMSs) emerge as crucial tools in smart buildings, balancing occupants’ comfort with Building Energy Efficiency (BEE) considerations^10^. The KNX system, an open global standard for building automation, offers energy consumption control and ease of programming, contributing to its widespread use and adaptability^7^.

In the residential sector, characterized by high energy demand, smart, wirelessly connected devices are explored to enhance energy efficiency and user convenience^11^. Dell’Isola et al.^12^ propose an approach to estimate device-specific energy consumption in energy monitoring IoT applications, contingent on household configuration and device information. Energy management systems operating on IoT principles, highlighted by^5^, reduce costs, improve efficiency, and facilitate intelligent control of lighting.

Efforts toward smart energy consumption management, according to^13^, not only have positive environmental impacts by reducing energy use but also contribute to sustainable practices and climate change mitigation. The ultimate goal, as emphasized by^14^, is to leverage smart technologies in buildings to achieve the lowest energy cost without compromising the environmental life cycle of the building. The amalgamation of these findings underscores the critical importance of exploring smart energy consumption management, particularly in the unique context of Palestine, as detailed in the study's objectives.

The examination of existing literature brings to light a substantial research gap in the realm of smart energy consumption management within Palestine, particularly in the context of residential domains. This discernible absence in scholarly exploration serves as the underpinning rationale for crafting hypotheses aimed at addressing the identified research void. The acknowledgment of this gap underscores the need for the current study to contribute meaningful insights and knowledge to an area that has, thus far, received limited attention in the academic discourse within the Palestinian context.

The research goals, outlined in consideration of the study’s significance, encompass various aspects related to smart energy consumption management systems, particularly in the context of residential buildings. The study's primary focus is on elucidating and showcasing the advantages of these systems, emphasizing potential benefits such as reduced energy usage, cost savings, and enhanced sustainability. Additionally, it endeavors to investigate the impact of educational and awareness initiatives on residents’ knowledge and behavior concerning smart energy consumption management, with the overarching goal of understanding how disseminating knowledge can foster more responsible energy use.

Furthermore, the study aims to identify and scrutinize the key factors influencing the successful implementation of smart energy management systems. This includes a thorough examination of IoT technology, energy-efficient equipment, and resident behavior, with a dual purpose of uncovering challenges and opportunities associated with these factors. Conducted with a specific focus on the West Bank, the research seeks to assess the feasibility and relevance of implementing smart energy consumption management systems in this particular regional context, aiming to determine their potential adoption and effectiveness.

In line with its comprehensive objectives, the study aspires to offer building owners and decision-makers valuable suggestions and guidance on supporting the integration of intelligent energy consumption management systems in the Palestinian residential sector. Ultimately, these efforts aim to contribute to the development of more effective and sustainable energy policies by enhancing understanding of the benefits of smart systems and the various factors influencing their successful adoption, particularly within the unique dynamics of the West Bank.

## Model conceptualization and hypothesis development

The concepts of modeling and hypothesis creation or generation are two closely related terms in the field of scientific research. Generally, the term conceptual modeling is about developing a theoretical framework or model to explain a particular phenomenon or group of phenomena, meanwhile, hypothesis generation involves creating testable specific data based on the previously mentioned model^15^.

### Conceptualization of the proposed model

**Figure 1:**
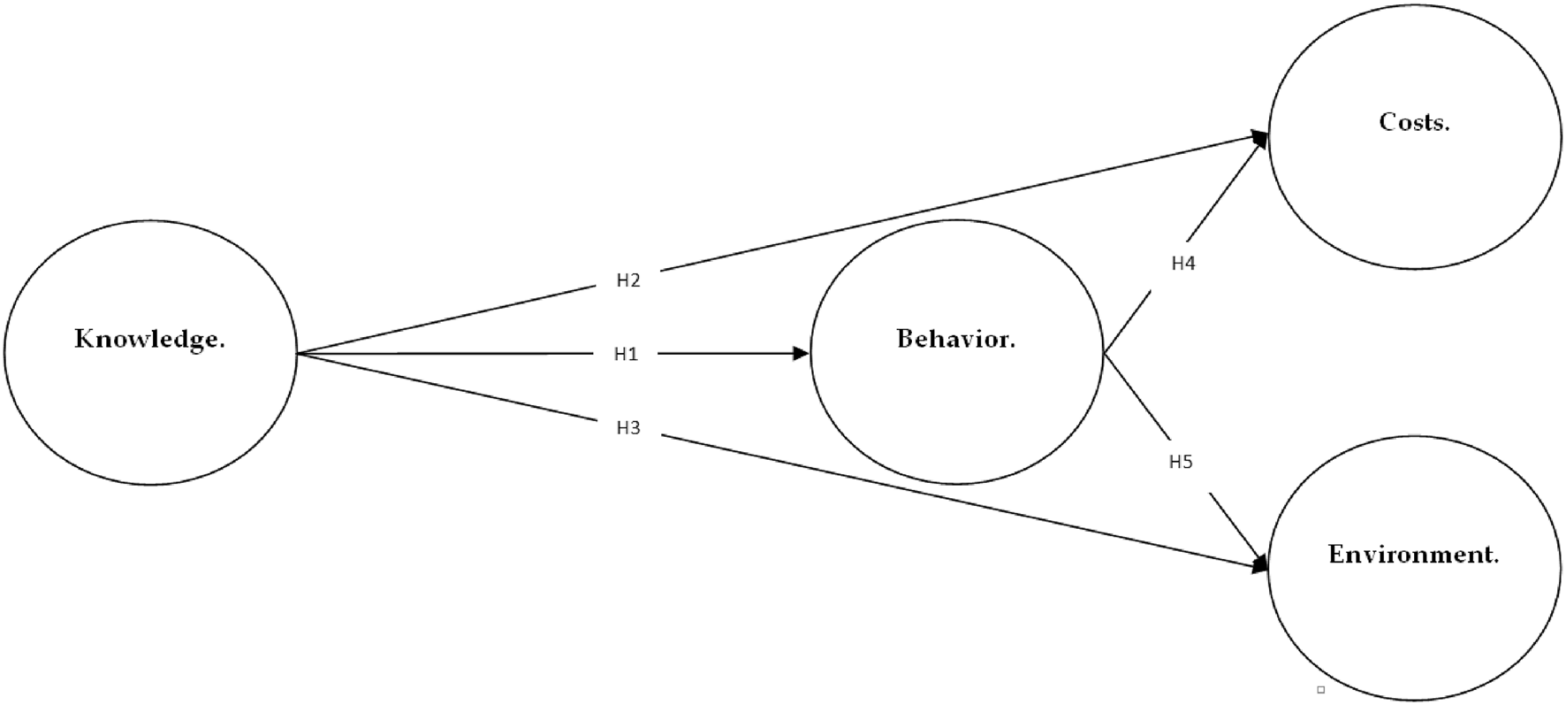
The research model and proposed hypotheses.

The primary objective is to establish a comprehensive understanding of the basic research model. This entails defining the scope and criteria of the model while also delving into the fundamental concepts and their interrelationships, drawing on insights from prior studies. The proposed model is shown in Fig. 1, which comprises four distinct constructs. Among these constructs, two are focused on the residents, namely knowledge and behavior. The remaining constructs pertain to the costs associated with smart energy consumption management in buildings, as well as the environmental impact of implementing such systems. It is important to note that all four constructs are presumed to possess reflective indicators, which will aid in evaluating their respective attributes and characteristics. The four constructs have a total of 13 indicators as discussed below, Table 1 summarized this operationalization of the model constructs.

**Table 1:** Operationalization of model constructs.

Construct	Item	Indicator	Reference
Knowledge	K1	Tasks	^9^
	K2	Time	^18^
	K3	Effort	^14^
	K4	IoT principals	^5^
	B1	Lightening	^19^
Behavior	B2	Conditioners	^20^
	B3	Heaters	^21^
	SCMC1	Energy consumption	^14,22^
Costs	SCMC2	Installation	^22^
	SCMC3	Type	^22^
	EE1	Sustainability	^14^
Environmental effect	EE2	Residential role	^23^
	EE3	Governmental role	^23^

### Knowledge

The residential sector is emerging as a high consumer of energy, necessitating exploration of wirelessly connected smart devices to enhance energy efficiency, user convenience, and overall quality of life^11^. Smart grid technologies enable intelligent and user-centric control, making it possible to integrate household devices for scheduling and administration. This promotes the concept of “demand-side intelligence,” providing consumers with real-time information on electricity costs and consumption to enhance their awareness and energy management, ultimately fostering a more sustainable residential energy culture^16^. Understanding smart energy consumption management systems is critical to selecting appropriate solutions based on building characteristics and measuring population awareness. This research delves into the residential knowledge related to smart energy consumption management in buildings, providing multiple vital insights. Primarily, the large energy consumption of the residential sector within buildings emphasizes the importance of energy management. Effective strategies in this sector can have a significant impact on energy conservation in general. Furthermore, as global energy and environmental concerns persist, optimizing energy use in buildings, particularly through smart energy management, is pivotal. This approach can reduce energy waste and greenhouse gas emissions, thus promoting a sustainable future. Moreover, investigation of residential knowledge in the context of intelligent energy consumption management can reveal obstacles to adopting energy-saving practices. Policy-makers and researchers can leverage this understanding to design effective interventions that facilitate energy-efficient behaviors and accelerate the shift towards a sustainable energy landscape. The study also emphasizes the importance of energy management tasks, including optimizing energy use, minimizing environmental impact, control mechanisms, and demand elasticity. As society grapples with energy and environmental challenges, understanding these tasks becomes essential, as their implementation enhances energy efficiency in buildings, particularly the residential field^9,17^. Efforts to reduce energy consumption and create comfortable building environments are simplified by resident knowledge. By increasing awareness of energy use patterns and offering customized solutions, residents can contribute to sustainable changes in energy management behaviors, leading to reduced consumption^14^. Smart management of energy consumption reduces installation and maintenance time, with the help of resident participation and customization^18^.This study provides insights into improved processes that require fewer resources and time. IoT principles play a pivotal role in energy efficient smart consumption management systems by enabling connectivity and automation. Understanding these principles, influenced by resident behavior, enables energy optimization strategies, and ultimately supports efficient, sustainable and cost-effective buildings^5^.

### Behavior

The impending increase in technological progress and population growth requires expanded energy production and investment in infrastructure. However, an alternative approach is to shift community behavior towards energy efficiency practices^11^. Understanding resident energy consumption patterns is critical to effective energy management. By analyzing behavior, wasteful practices such as unnecessary lighting or using appliances at peak times can be reduced, reducing energy waste and promoting sustainability. Educating residents about energy conservation and providing them with tools promotes a culture of energy efficiency, which leads to significant savings and reduced emissions.

Smart lighting systems, which evolve with sensor input and user data, offer improved energy savings and functionality^19^. Behavior recognition allows for customized lighting solutions, behavior change incentives, and occupancy-based optimization, resulting in environmentally friendly, customized, and comfortable buildings. In air conditioning, energy-saving strategies, such as demand-response control, deal with peak loads^20^. Behavior recognition enables energy-efficient temperature control, occupant satisfaction, and adjustments based on external conditions, resulting in sustainable and comfortable environments. Water heating, a major energy consumer, sees developments in hybrid solutions^21^. Insights into resident behavior enable opportunities for energy savings, personalized temperature control, and resident comfort, leading to increased energy efficiency. In all cases, understanding behavior enables targeted solutions, reducing consumption, promoting sustainable practices, and ultimately enhancing efficiency, population satisfaction, and environmental well-being.

### Cost

Cost investigation in the field of smart energy management yields multifaceted benefits, including cost reduction, environmental impact mitigation, and enhanced energy security. This endeavor fosters efficiency enhancements, stimulates innovation, and fuels economic growth, thus underpinning a sustainable and secure energy framework. The IoT embodies efficiency, convenience, and cost-effectiveness, shaping contemporary living standards^22^. Smart energy management systems, by profiling and predicting energy consumption, enable timely response and consumption reduction^14^. Scrutinizing the costs of these systems leads to significant energy savings through various methods. First, identifying inefficiencies enables stakeholders to upgrade aging equipment, enhancing energy efficiency. Second, real-time monitoring detects unnecessary consumption, which motivates behavior change. Finally, information about cost motivates energy reduction efforts. Overall, delving into the costs of smart energy consumption drives a sustainable future by reducing energy use and emissions.

Cost assessment greatly influences the installation of an energy management system. It improves solution selection, addresses barriers to adoption, and drives innovation. Informed decisions about system selection, considering installation and maintenance costs, lead to effective results. Policymakers, investors, and companies can collaboratively overcome hurdles to adoption, based on insights from cost analysis. The complexities of system costs depend on a variety of factors, including building size, depth of automation, and interaction between hardware and software^22^. Moreover, system differentiation imposes cost differences, with comprehensive control systems incurring higher expenses compared to single-function systems such as lighting or air conditioning^22^. Cost-driven exploration of intelligent energy consumption management helps in selecting appropriate types of energy management, which meet the building's specific needs. By distinguishing cost efficiencies and potential savings across system types, stakeholders are empowered to choose optimal solutions for energy reduction and financial conservation.

### Environmental effect

Energy professionals, including engineers, planners, designers and researchers, are actively integrating smart technologies into buildings with the overarching goal of lower energy cost and minimal environmental impact across the building lifecycle. The potential of smart technologies to improve energy efficiency, reduce waste and raise the level of sustainability in construction operations is recognized. Their aim is to align economic viability and environmental accountability in the pursuit of sustainable development^14^.

Central to this endeavor is sustainability, a pivotal consideration in the design of energy consumption management systems for smart buildings. These systems aim to optimize energy use and reduce losses, thus reducing operational expenses and achieving positive environmental outcomes. The transformation of traditional buildings into smart energy structures depends on the adoption of smart energy technologies, which leads to reduced consumption and more efficient energy management^14^. Residents’ participation is key to the victory of smart energy consumption management systems. By adopting energy-saving behaviors and using the tools provided by the system, occupants can make a significant contribution to energy conservation. Recognizing that behavior modification in low-cost activities and minor structural modifications can lead to significant energy savings, an understanding of resident energy conservation behavior is essential. This understanding informs efficient energy policies tailored to residents’ needs and encourages home energy savings^23^. Government participation also plays an important role in developing smart management of energy consumption in buildings. By formulating policies, regulations and financial incentives, governments can promote energy-saving technologies and adopt smart consumption management system. Insights into population needs and preferred behavioral changes guide the government in developing energy saving strategies and guidelines for households^23^.

In this research model, various constructs related to smart energy consumption management in buildings are defined and operationalized. These constructs help to understand the different aspects of smart energy consumption management and its implications on energy efficiency, user behavior, and environmental impact. Each construct is associated with specific items and indicators to measure and analyze the relevant variables. The following table is a summarization of each construct and its indicators with the references cited from.

### Hypothesis development

After Following an exhaustive review of existing literature, a profound comprehension of the scope and insufficiencies in smart energy consumption management in residential contexts was established. This exploration unearthed a significant research void within Palestine concerning smart energy consumption management, specifically in residential domains. This gap became the bedrock for formulating hypotheses that address the aforementioned research void. Hypothesis formulation encompasses positing research questions and proposing provisional explanations or predictions that subsequent empirical research can scrutinize.

The first hypothesis (H1) postulates that residents possessing knowledge of smart energy consumption management systems will exhibit greater adherence to sustainable energy behaviors. Hypothesis 2 (H2) suggests that residents’ awareness of smart energy consumption management correlates positively with reduced costs associated with such systems. Hypothesis 3 (H3) asserts a positive link between residents’ awareness and a decrease in environmental impact attributed to smart energy consumption management systems. Hypothesis 4 (H4) posits that adjective resident behavior within buildings engenders cost reduction linked to smart energy consumption management systems. Hypothesis 5 (H5) suggests that positive resident behavior also leads to a diminution of potential environmental harm stemming from energy consumption practices. These hypotheses collectively constitute a framework for systematic exploration and evaluation of the interplay between resident knowledge, behavior, costs, and environmental impact in the context of smart energy consumption management within residential buildings.

## Methodology

The research process encompasses formulation, execution, and analysis stages. Formulation involves defining the problem, literature review, setting objectives, formulating hypotheses, and designing the study. Execution selects sampling, data tools, collection, and storage. The analytical phase encompasses data analysis, hypothesis testing, interpretation, and deriving conclusions.

**Figure 2:**
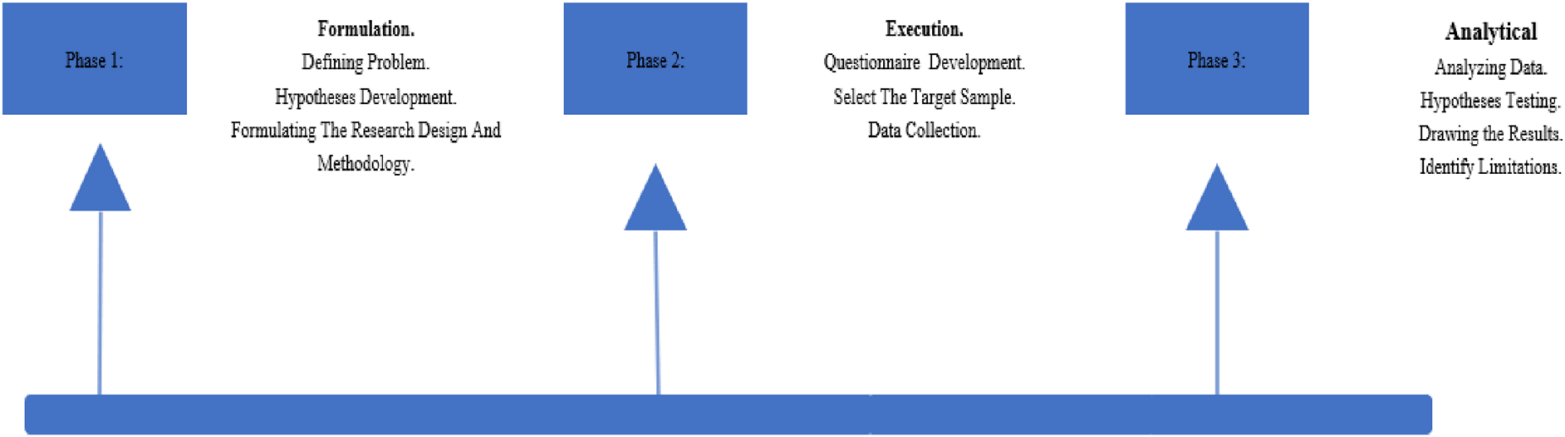
Research methodology.

The methodology follows three stages, initiating with defining the research problem—a focus on residents’ role in sustainable smart energy consumption, including behavior impact. Hypotheses stem from literature review, adopting a mixed research approach. Stage two involves questionnaire design, vetted by experts, targeting 100 valid responses from the West Bank via social media. Collected data underwent analysis using smart-PLS, affirming hypotheses, and generating results, discussion, and recommendations. Figure 2 illustrates the research flow chart in this study.

### Design of the research

This research is carefully designed to be bears the characteristics of the quantitative research. An online survey was designed to collect residential opinions for testing the hypotheses of the research model. The online survey was structured into five main sections. The initial section focused on gathering general information about the respondents' buildings, such as the province, building size, monthly electricity bill, and whether it had a traditional or smart system. The second component of the survey asked questions about inhabitants’ knowledge and comprehension of smart energy management in buildings. In the third portion, broad questions about inhabitants' eating habits at home were asked. In the fourth segment, the cost of deploying smart technology for energy management was intended to be discussed with locals. The fifth and final segment looked at the effects that these systems’ implementation had on the environment. A 5-point Likert scale was used in the final four sections to rate responses. The four components of the study model's four multi-item reflecting measures were created using the questions.

### Data collection analysis tools

The survey's primary goal was to gather data for use in evaluating the constructs' hypotheses. This data was analyzed using SmartPLS software, which is particularly well-suited for lower sample sizes in comparison to other methodologies, with 100 valid replies. PLS is renowned for its resilience, capacity to manage assumptions violations, and superior performance with lower sample sets. Due to its efficacy in assessing intermediate variables and indirect links, as well as its strong capacity to examine sample sizes, Partial Least Squares-Structural Equation Modeling (PLS-SEM) was applied in this study to test our hypotheses. The suggested model’s validity and dependability may also be directly tested using the Smart Partial Least Squares (SmartPLS) program.

### Ethical statement

This online survey study has been conducted in strict adherence to established ethical principles and guidelines. We have obtained informed consent from all participants, maintained their privacy and confidentiality, ensured data security, and minimized any potential harm or discomfort to participants. The study has also received approval from our institution's ethics committee. We are committed to using the collected data solely for research purposes and will protect the anonymity of all participants.

## Analysis and results

In order to find patterns, correlations, and trends that may be utilized to influence decisions, the data gathered via research will be analyzed and interpreted. Our goal is to assess research results in order to support judgments and decisions. Large volumes of quantitative data must be gathered, arranged, and interpreted during the analysis stage utilizing a number of techniques such descriptive statistics, illustrations, and visualizations. In this step, the questionnaire results were assessed, and the model's validity and reliability as well as the validity of the hypotheses were evaluated using the SmartPLS4 software.

### Questioner analysis

To analyze data and find links between variables, Smart PLS use a number of graphical and statistical approaches, including mechanical equation display and variance-based structural equation modeling. This approach can help us understand which factors are most crucial and how they interact, enabling us to develop strategies and take better informed decisions. Additionally, Smart PLS enables automatic model parameter estimation and offers the capability of swiftly and simply evaluating various measurement models. developing and testing the quantity model comes first, tracked by developing and testing the structural model. This two-step technique will be used to conduct the analysis.

### Evaluation of the measurement model (outer model)

The examination of the reflective quantity model is seen to be essential for determining the validity and dependability of the construct. The term "dependability" in the context of the reflective quantity model refers to the reliability and consistency of the construct being measured. It encompasses the ability of the measurement model to accurately and consistently capture the intended concept under various conditions. In other words, dependability indicates the extent to which the construct can be trusted to yield consistent and valid measurements across different situations and contexts. This includes the reliability of the measurement items and their connections to the latent variables, as well as the overall stability and robustness of the construct’s representation within the model. Essentially, dependability ensures that the construct is accurately and consistently measured, providing a reliable basis for analysis and interpretation within the research framework. The reliability is the ability to measure what is required, and the validity is the ability to measure what is required under several different circumstances.

The assessment model, which consists of the measurement items and their connections to the corresponding latent variables, was assessed using the PLS-SEM method^20^. Convergent validity tests and discriminating validity tests are part of the evaluation of the measurement model.

#### Convergent validity

Convergent validity describes how closely many measurements of a single concept relate to one another. The factor loadings of the gauges on their respective latent variables are one of the basic techniques used to evaluate convergent validity. The metric that captures the structural equation model's strongest point in the link between the indicator and the underlying construct is known as factor packing. Factor packing refers to the metric that captures the structural equation model's strongest point in the link between the indicator and the underlying construct. In other words, it indicates the amount of variance in the observed variable (i.e., the indicator) that is explained by the underlying construct. This concept is crucial in understanding the strength of the relationship between the observed variables and the latent constructs in a structural equation model. Essentially, factor packing provides insights into how well the indicators represent the underlying constructs, shedding light on the robustness and reliability of the measurement model^24^.

When relying on factor packing to assess convergent validity, it is important to acknowledge potential limitations and considerations. Factor packing, which measures the amount of variance in the observed variable explained by the underlying construct, can be influenced by factors such as multicollinearity and the presence of outliers in the data. Multicollinearity, where independent variables are highly correlated, can impact the accuracy of factor loadings and subsequently affect the assessment of convergent validity. Additionally, outliers in the data can disproportionately influence the variance explained by the underlying construct, potentially skewing the results of factor packing. Therefore, it is essential to carefully consider these factors and their potential impact when utilizing factor packing as a measure of convergent validity. This ensures a comprehensive and nuanced evaluation of the measurement model.

Convergent validity, a pivotal aspect of our study, is assessed using the Average Variance Extracted (AVE) method. AVE values exceeding 0.5, a common criterion, indicate satisfactory convergent validity^25^. Each construct in our research model exhibits AVE values surpassing 0.5, affirming strong association between indicators and intended constructs. Additionally, Composite Reliability (CR) confirms internal consistency; values above 0.7 are acceptable^25^. All model constructs and Cronbach's Alpha values exceed this threshold, affirming reliable measurement as shown in Table 2. Composite Reliability, particularly suited for models with fewer constructs, also exceeds 0.7^25^. However, these measures must be considered alongside other validity and reliability assessments for a comprehensive evaluation of our PLS-SEM analysis. Discriminatory validity, reliability, and model fit are also vital considerations in overall quality assessment.

**Table 2:** Construct reliability and validity.

Construct/method	Cronbach’s alpha	CR	AVE
Knowledge	**0.852**	**0.858**	**0.693**
Behavior	**0.817**	**0.824**	**0.732**
Cost	**0.807**	**0.808**	**0.721**
Environmental effect	**0.792**	**0.797**	**0.707**

Significant values are in (bold).

#### Discriminant validity

Discriminant validity is a concept that refers to the degree to which the constructs being measured in a study are distinct from one another. In other words, it measures whether two constructs are measuring the same thing or are truly distinct^25^. Discriminant validity is important in research because if two constructs are not distinct from each other, then it can lead to inflated correlations and affect the validity of the results. In this context, discriminant validity can be assessed using several methods.

The Fornell-Larcker criterion is a widely used method for assessing discriminant validity, which measures the extent to which constructs in a study are distinct from one another. This criterion compares the square root of the Average Variance Extracted (AVE) of each construct to the correlations between constructs. If the AVE of a construct exceeds the correlation coefficients with other constructs, it indicates that the construct has discriminant validity, meaning it is distinct from other constructs being measured. In the context of the study, the Fornell-Larcker criterion is used to ensure that the constructs, such as knowledge, behavior, cost, and environmental effect, are distinct and not measuring the same underlying concept, thus affirming the validity of the measurement model^25^. Typically reported, Fornell-Larker values in Table 3 show AVE on the diagonal and correlations off-diagonal. In the context of the study, the Fornell-Larcker criterion was employed to ensure that the constructs, such as knowledge, behavior, cost, and environmental effect, are distinct and not measuring the same underlying concept, thus affirming the validity of the measurement model. This criterion is widely used in structural equation modeling to assess the distinctiveness of constructs and is an essential component of discriminant validity assessment. Our study's AVE values exceed construct correlations, affirming discriminant validity (e.g., Knowledge’s AVE of 0.833 > Behavior: 0.814, Cost: 0.790, Environmental Effect: 0.804).

**Table 3:** Latent variable correlation.

Construct	Knowledge	Behavior	Cost	Environmental effect
Knowledge	**0.833**			
Behavior	0.814	**0.856**		
Cost	0.790	0.799	**0.849**	
Environmental effect	0.804	0.803	0.795	**0.841**

Significant values are in (bold).

Assessing discriminant validity also involves Cross-loadings, which gauge if an indicator aligns with its intended construct. Cross-loading examines how each item loads on its construct versus others. In Table 4, indicators’ placements relative to constructs are shown. This approach ensures discriminatory validity. Results in Table 4 offer cross-loading parameter values, reflecting indicator loads within each construct. For instance, examining Behavior’s column reveals that all its indicators (e.g., B1, B2, B3) load more on Behavior than other constructs, meeting the criterion. In the case of Behavior, factor loadings are (0.902), (0.832), and (0.832) while (0.761), (0.663), (0.660) with Knowledge, (0.731), (0.680), and (0.635) with Cost, and (0.752), (0.702), and (0.600) with Environmental Effect, solidifying discriminant validity.

**Table 4:** Cross loading.

Indicator/construct	Knowledge	Behavior	Cost	Environmental effect
K1	**0.832**	0.639	0.629	0.637
K2	**0.830**	0.729	0.718	0.713
K3	**0.887**	0.719	0.704	0.711
K4	**0.778**	0.614	0.568	0.609
B1	0.761	**0.902**	0.731	0.752
B2	0.663	**0.832**	0.680	0.702
B3	0.660	**0.832**	0.635	0.600
SCMC1	0.658	0.744	**0.867**	0.693
SCMC2	0.709	0.612	**0.836**	0.628
SCMC3	0.649	0.675	**0.844**	0.704
EE1	0.597	0.637	0.648	**0.810**
EE2	0.698	0.685	0.663	**0.837**
EE3	0.726	0.702	0.693	**0.873**

Significant values are in (bold).

**Figure 3:**
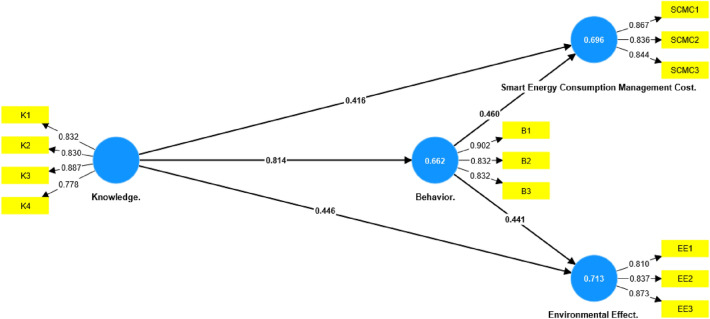
Model framework—PLS-SEM algorithm.

Figure 3 presents a set of information that resulted from the PLS-SEM algorithm analysis on the model framework, such as the factor loadings that appears between the indicators and constructs in addition to the path coefficient which is the estimated strength and direction of the relationship between two constructs in a structural model^26^.

By analyzing convergent validity, it was found that the measurement model fulfilled this test in all analytical respects.

### Assessment of structural model (inner model)

The links between the latent variables themselves are described by the structural model. The path coefficients, which show the strength and direction of the association between each pair of latent variables, are specified to do this^25^. These various statistical measures to evaluate the model fit, and at this section, we will shed light on some these methods.

#### Testing hypothesis

**Figure 4:**
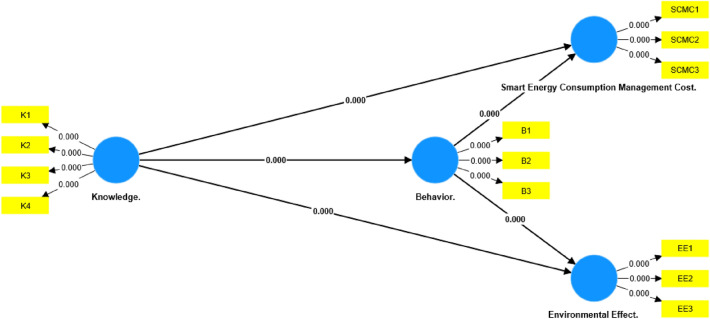
Model framework—*P*-values.

**Figure 5:**
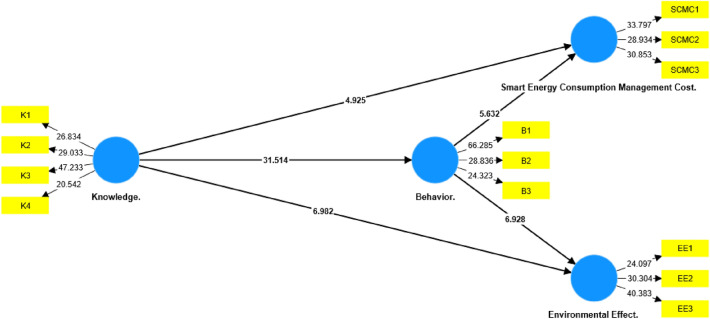
Model framework—T-statistics.

Testing The assessment of hypotheses in SmartPLS entails a statistical analysis encompassing the structural model, including measurement and construct relationships^21^. Significance of latent variable relationships relies on path coefficients equal to or exceeding 0.1 (Fig. 3). The t-value’s importance, gauged by ≥ 1.96 at a 5% significance level, was evaluated. Bootstrapping, modified to a 0.05 significance level with 100 iterations (Table 5), and analysis of *P*-values and T—Statistics for indicators and constructs (Figs. 4 and 5) were conducted for comprehensive model assessment (Fig. 5).

**Table 5:** Path coefficient, STDEV, T values, and *P*-values.

Hypothesis/Criteria	Path coefficient	Standard deviation (STDEV)	T statistics (|O/STDEV|)	*P*-values
Knowledge.—> Behavior	**0.814**	**0.026**	**31.514**	**0.000**
Knowledge.—> Cost	**0.416**	**0.084**	**4.925**	**0.000**
Knowledge.—> Environmental effect	**0.446**	**0.064**	**6.982**	**0.000**
Behavior.—> Cost	**0.460**	**0.082**	**5.632**	**0.000**
Behavior.—> Environmental effect	**0.441**	**0.064**	**6.928**	**0.000**

Significant values are in (bold).

In structural equation modeling (SEM), path coefficients play a crucial role in revealing the strength and direction of relationships between latent variables, akin to how regression coefficients are interpreted. Firstly, the sign of the path coefficient, whether positive or negative, signifies the direction of the relationship between variables. A positive coefficient denotes a positive relationship, indicating that an increase in the independent variable corresponds to an increase in the dependent variable, while a negative coefficient suggests an inverse relationship, where an increase in the independent variable leads to a decrease in the dependent variable.

Secondly, the magnitude of the path coefficient conveys the strength of the relationship between variables. A larger coefficient indicates a more robust relationship, whereas a smaller coefficient implies a weaker connection. To gain insights into the relative strengths among relationships within the model, one can compare the magnitudes of different coefficients.

Table 5 presents compelling statistical evidence for the hypotheses tested. The *P*-value of 0.000 for H1 signifies the strong rejection of the null hypothesis, revealing that residents with knowledge of smart energy consumption management are inclined towards adopting sustainable energy behaviors, aligning with the qualitative interview findings. Similarly, H2’s *P*-value of 0.000 underscores the link between residents' knowledge of smart energy systems and reduced costs, in harmony with interviews. For H3, a *P*-value of 0.000 affirms the positive correlation between knowledge and diminished environmental impact, consistent with both quantitative and qualitative outcomes. H4’s *P*-value of 0.000 validates the assertion that residents' good behavior correlates with lower costs, carrying implications for building management. Lastly, H5’s *P*-value of 0.000 indicates robust evidence supporting the notion that residents' positive behavior curtails environmental harm, underscoring the pivotal role of resident behavior in environmental preservation.

#### Coefficient of determination (R-squared values)

The coefficient of determination (R^2^) gauges the extent to which independent variables account for variation in the dependent variable^25^. Table 6 presents R^2^ values for study constructs, measuring model’s explanatory power. For dependent variables (Knowledge, Behavior), R^2^ values exceeding 0.6 indicate robust model agreement—highlighting effective independent variable influence. However, context and theoretical relationships also impact interpretation, urging examination of additional fit measures and variable connections. Chin’s (1998) interpretation criteria indicate that an R-Square value of 0.67 or higher is considered good, while values between 0.33 and 0.67 are considered moderately good, and values less than 0.33 are considered less satisfactory^25^.

**Table 6:** Quality criteria—R^2^ and Q^2^.

Construct	R-square	Q^2^ predict
Behavior	0.662	0.653
Cost	0.713	0.618
Environmental effect	0.696	0.640

#### Predictive relevance (Q—squared values)

Predictive relevance is commonly evaluated with Q^2^, which gauges latent variables' ability to predict endogenous variables^25^. Q^2^ is derived via cross-validation, estimating model parameters on a subset and evaluating prediction on the rest. High Q^2^ signifies robust predictive power, while low values suggest variable scrutiny. Table 6 presents Q^2^ predict values, indicating structural model's predictability. A Q^2^ value exceeding 0.6 suggests strong predictive capability, though interpretation hinges on context and research focus. Other strengths, like fit and path coefficients, also impact assessment. Q^2^ must be considered alongside validation indices, effect sizes, and significance levels for comprehensive model evaluation. A Q^2^ value of 0.25 or higher is considered good, while values between 0.15 and 0.25 are considered moderately good, and values less than 0.15 are considered less satisfactory^25^.

#### Effect size (F-squared values)

Effect size F-Square quantifies variance explained by an independent variable in relation to a dependent variable, while Standardized Root Mean Square Residual (SRMR) gauges observed-predicted correlation disparities. Table 7 presents effect size values for study constructs. In our study, F^2^ values are employed to scrutinize predictor variables within the structural equation model, specifically indicating the proportion of variance accounted for by a predictor variable after accounting for other predictors. Cohen’s 1987 criteria categorize F^2^ values: 0.02 (small), 0.15 (medium), and 0.35 (high) effect sizes. In the context of the study, small, medium, and high effect sizes (0.02, 0.15, and 0.35, respectively) are used to interpret the impact of predictor variables on the dependent variables. These effect sizes provide a measure of the proportion of variance in the dependent variable that can be explained by the independent variable, after accounting for other predictors.

**Table 7:** Effect size (F^2^).

	Behavior	Cost	Environmental effect
Knowledge	1.963	0.192	0.233
Behavior		0.235	0.228

A small effect size (0.02) indicates that the independent variable accounts for a small proportion of the variance in the dependent variable. This suggests a relatively weak relationship between the predictor and the outcome. A medium effect size (0.15) signifies a moderate proportion of variance in the dependent variable explained by the independent variable. This suggests a more substantial relationship between the predictor and the outcome, indicating a moderate level of influence. A high effect size (0.35) indicates that the independent variable accounts for a large proportion of the variance in the dependent variable. This suggests a strong and influential relationship between the predictor and the outcome.

In the study, these effect sizes impact the interpretation of the relationships between predictor variables by providing a quantitative measure of the strength of these relationships. By considering the effect sizes alongside other statistical measures such as R^2^, path coefficients, and P-values, researchers can comprehensively understand the impact of predictor variables on the dependent variables. This allows for a nuanced interpretation of the relationships and their significance in the context of the study^25^.

#### Goodness of fit index (GoF)

The structural model can be evaluated in several ways to ensure its accuracy and reliability so that the structural model can be evaluated using the quality of fit indicators. These indicate the fit of the representative to the experimental data. In addition, it can be evaluated by comparing the results of the model with an independent test set.

Goodness of fit index (GoF) which in turn means the ability to rely on the proposed model, whether on the measurement model represented by the AVE and structural model represented by the R^2^. it can be calculated through a simple equation, which is:

Handing the square root of R^2^ average multiplied by the AVE average which can be written in Eq. (1) as follows:

1 $${\text{GoF Index }} = { }\sqrt {\overline{{{\text{AVE}}}} \,{*}\,\overline{{{\text{R}}^{2} }} } { }$$

By going back to the tables related to the previous equation, i.e., Table 2 and Table 6, we can calculate the general rate of excess and the general rate of deficiency and apply Eq. (1) to show the results as follows:

$$\overline{{{\text{AVE}}}} = \frac{{\left( {0.693{ } + { }0.732{ } + { }0.721{ } + { }0.707} \right)}}{4} = 0.713$$

$$\overline{{{\text{R}}^{2} }} = \frac{{\left( {0.662 + 0.713 + 0.696} \right)}}{3} = 0.690{ }$$

And by applying Eq. (1):

$${\text{GoF Index }} = { }\sqrt {0.713{*}0.690} = 0.701$$

A GoF index value of 0.701 in our search model indicates that the model has a moderate fit to the data. It should be emphasized that the GoF index, whose value goes from 0 to 1, was used to gauge how well the model fit the data observed, and that any higher values indicate a better fit. An adequate degree of fit is indicated by a result of 0.701, which shows that the model accounts for around 70.1% of the variation in the data. It is crucial to remember that the complexity of the model and the size of the sample affect how the GoF index should be interpreted. Along with carefully evaluating the model’s assumptions and constraints, it's crucial to take into the theoretical and practical importance of the results.

#### Mediation analysis

The Behavior variable, which is dependent on Residents’ Knowledge but independent of Cost and Environmental Effect, serves as a mediator in this model. Variables are classified as follows: Independent—Residents’ Knowledge, Dependent—Cost and Environmental Effect, Mediator—Residents' Behavior. Using mediation analysis, Preacher and Hayes (2008) present a frequently used methodology. The Preacher and Hayes mediation model is a widely used methodology for examining the mediating role of a variable in a relationship between an independent variable and a dependent variable. It involves testing the direct and indirect effects of the independent variable on the dependent variable through the mediator.

Bootstrapping is employed in this context to test the indirect effects of residents' knowledge on the cost of smart energy consumption management and environmental impact through behavior. It aids in testing indirect effects by resampling the data to generate an indirect effect distribution. This approach allows for the estimation of confidence intervals for the indirect effects, providing a more accurate assessment of the mediation of the behavior construct. By using bootstrapping, the study can assess the significance of the indirect impact and determine the strength of the relationships between the variables.

The three central hypotheses of this strategy, also known as the “Preacher and Hayes mediation model” or “bootstrapping method,” are that Knowledge affects Behavior (path a), Behavior affects Cost and Environmental Effect while controlling for Knowledge (path b), and the Knowledge-Cost/Environmental Effect relationship weakens or disappears with Behavior inclusion (path c). For the purpose of examining the indirect impact of knowledge on cost and environmental impact through behavior, we used bootstrapping to test these hypotheses. Bootstrapping entails resampling to generate an indirect effect distribution. Findings in Tables 8 and 9 affirm these hypotheses through path coefficient values and *P*-values.

**Table 8:** Indirect effects.

Relation	Path coefficient	Standard deviation (STDEV)	T statistics (|O/STDEV|)	*P*-values
Knowledge.—> Behavior.—> Cost	0.374	0.068	5.502	0.000
Knowledge.—> Behavior.—> Environmental effect	0.359	0.054	6.700	0.000

**Table 9:** Total effects (Mediation).

Relation	Path coefficient	Standard deviation (STDEV)	T statistics (|O/STDEV|)	*P* values
Knowledge.—> Behavior	0.814	0.026	31.514	0.000
Behavior.—> Cost	0.460	0.082	5.632	0.000
Behavior.—> Environmental effect	0.441	0.064	6.928	0.000
Knowledge.—> Cost	0.374	0.068	5.502	0.000
Knowledge.—> Environmental effect	0.359	0.054	6.700	0.000

Tables 8 and 9 present important findings from the study. Table 8 provides information on the path coefficients, standard deviations, t-statistics, and *P*-values for the relationships between the constructs. The path coefficients indicate the strength and direction of the relationships, while the t-statistics and *P*-values show the significance of these relationships. The results show strong and significant relationships between knowledge, behavior, costs, and environmental impact, supporting the study’s hypotheses.

Table 9 focuses on total effects, particularly in the context of mediation. It provides insights into the indirect effects of knowledge and behavior on costs and environmental impact. The results demonstrate the mediating role of behavior in the relationship between knowledge and the outcomes of cost and environmental effect. This highlights the importance of behavior as a key mediator, connecting knowledge to tangible outcomes in the context of smart energy consumption management in residential buildings.

Utilizing the confidence interval (CI), which represents a plausible range for the real population parameter, the significance of the indirect impact was evaluated. In this case, the parameter of interest is the indirect effect of residents' knowledge on the cost of smart energy consumption management and environmental impact via energy-saving behaviors. The bootstrap method computed CI for the indirect effect by resampling and calculating it across many new samples, yielding a distribution of possible values. Table 10 presents bias-corrected and accelerated (BCa) CI for indirect effects, offering improved accuracy by addressing bias and skewness. The non-inclusion of zero in the CI at a specified significance level (0.05) signifies statistical significance, affirming mediation of the behavior construct.

**Table 10:** Confidence intervals bias corrected.

Relation	Original sample (O)	Bias	2.5%	97.5%
Knowledge.—> Cost	0.374	0.010	0.252	0.499
Knowledge.—> Environmental effect	0.359	0.001	0.228	0.450

#### Variance inflation factor (VIF)

Understanding Variance Inflation Factor (VIF) values is crucial for assessing multicollinearity in a model. VIF values are consistently positive, representing the ratio of variance in a model with multiple predictors to that in a model with a single predictor. This measure indicates the extent of correlation among latent variables and is essential for ensuring the reliability of parameter estimates in structural equation modeling^25^. Guidelines for VIF interpretation suggest that values below 5 are generally acceptable, indicating a low to moderate level of multicollinearity. Values between 5 and 10 may signal a moderate to high level of multicollinearity, requiring careful consideration, while values exceeding 10 raise concerns about the precision of parameter estimates^25^.

In Table 11, A VIF value near 1.000 between “Knowledge” and “Behavior” allays concerns of multicollinearity, supporting the hypothesis that residents with knowledge about smart energy consumption adopt sustainable energy behaviors. Moderate VIF values between “Knowledge” and “Cost” suggest some multicollinearity. Cross-verification with additional diagnostics is advisable to understand its impact on the relationship between knowledge and cost reduction. Similar to H2, moderate VIF values between “Knowledge” and “Environmental Effect” suggest potential multicollinearity. Comprehensive diagnostics should be applied for a robust interpretation of the relationship. A VIF values between “Behavior”, “Cost” and “Behavior”, “Environmental Effect” may indicate modest correlation but remain within an acceptable range, supporting the hypothesis that good resident behavior positively impacts cost reduction and minimizing environmental damage.

**Table 11:** Variance inflation factor (VIF).

Hypothesis	VIF
Knowledge.—> Behavior	1.000
Knowledge.—> Cost	2.963
Knowledge.—> Environmental effect	2.963
Behavior.—> Cost	2.963
Behavior.—> Environmental effect	2.963

While moderate multicollinearity may not invalidate hypotheses, it could impact the precision of coefficient estimates. Augment VIF assessments with complementary diagnostic measures like tolerance values, eigenvalues, or condition indices for a comprehensive evaluation. If concerns persist, consider strategic adjustments such as removing redundant variables or conceptual consolidation to enhance model stability.

In conclusion, the VIF values, while signaling some multicollinearity, align with acceptable ranges. A meticulous analysis, considering various diagnostics, is paramount for nuanced interpretation in line with research objectives and hypotheses. Always refer to relevant statistical guidelines for informed decision-making in structural equation modeling.

## Discussion

SmartPLS is particularly well-suited for lower sample sizes due to its resilience and capacity to manage assumption violations. For example, in the context of lower sample sizes, SmartPLS can effectively handle situations where traditional statistical methods may not be applicable. It does so by employing a non-parametric bootstrapping technique, which allows for the estimation of standard errors and confidence intervals without relying on the assumption of normality in the data. This is especially beneficial when dealing with smaller sample sizes, as it provides robust estimates and enhances the reliability of the results.

Additionally, SmartPLS is adept at managing assumption violations, such as non-normality and non-linearity in the data. It does not require strict adherence to assumptions of multivariate normality, making it more flexible and applicable to real-world data, which often deviates from ideal assumptions. By employing a variance-based structural equation modeling approach, SmartPLS is able to handle complex relationships and non-linearities in the data, providing a more accurate representation of the underlying constructs and their interrelationships.

However, it is important to acknowledge that while SmartPLS offers several advantages, there are also considerations and limitations associated with its use. For instance, the interpretation of results in SmartPLS requires careful consideration, as it may not provide as detailed insights into the underlying distributional properties of the data compared to other statistical methods. Additionally, the choice of methodology should align with the specific research objectives and the nature of the data being analyzed. It is essential to recognize that no single method is universally superior, and researchers should carefully weigh the strengths and limitations of SmartPLS in the context of their research goals and data characteristics.

In the discussion section, the study delves into the intricate connections among smart energy knowledge, resident behavior, and the outcomes of cost and environmental impact. The research employed PLS-SEM analysis to test five hypotheses, and the results were significant. The study found that knowledge of smart energy consumption management systems influences the adoption of sustainable energy behaviors, leading to reduced costs and a positive impact on the environment. The study also established a connection between good behavior and lowered costs through enhanced energy efficiency, as well as the pivotal role of resident behavior in minimizing environmental damage.

These findings were supported by mediation analysis results, which included bootstrap-derived confidence intervals, path coefficient values, and *P*-values. The study underscores the need for a comprehensive approach encompassing education, feedback mechanisms, and policy interventions to foster sustainable energy practices. This holistic strategy, vital for promoting cost-effectiveness, energy conservation, and environmental sustainability, must be embraced by both decision-makers and the wider populace.

The study's results provide invaluable insights to policy makers and stakeholders, enabling the formulation of effective strategies to increase energy efficiency and reduce the environmental burden of energy consumption in the residential sector in Palestine. The research also sheds light on the relationship between residents’ knowledge and behavior within homes, and their implications for costs and the environment. Additionally, the study emphasizes the importance of integrating cutting-edge technologies like IoT and AI to optimize energy consumption and minimize environmental harm.

## Conclision

The conclusion section of the document provides a comprehensive overview of the study's findings and their implications. It highlights the complex relationship between knowledge, behavior, costs, and environmental sustainability in smart energy management for residential buildings. The study utilized mediation analysis to demonstrate behavior as a key mediator, connecting knowledge to tangible outcomes. The findings revealed that the dissemination of information can lead to changes in resident behavior, resulting in reduced expenses and a positive impact on the environment. The study acknowledges its limitations, such as context-specificity and reliance on self-reported data, and encourages further research to explore cross-cultural dynamics and objective measures.

In terms of future work, the study suggests the need for further research to examine cross-cultural dynamics and objective measures. It also emphasizes the importance of a comprehensive approach involving education, feedback, and effective policies to achieve sustainable results. The study encourages the integration of cutting-edge technologies like IoT and AI to optimize energy consumption and minimize environmental harm. Additionally, the document underscores the practical implications of the research, providing valuable insights for policymakers and stakeholders to formulate effective strategies for increasing energy efficiency and reducing the environmental burden of energy consumption in the residential sector in Palestine.

The theoretical implications of the study lie in its contribution to addressing the research gap within Palestine concerning smart energy consumption management, specifically in residential domains. The study's framework and hypotheses provide a systematic exploration and evaluation of the interplay between resident knowledge, behavior, costs, and environmental impact in the context of smart energy consumption management within residential buildings. Furthermore, the study’s theoretical implications extend to the broader academic discourse, enriching the understanding of the relationship between residents' knowledge and behavior within homes, and their implications for costs and the environment.

## Data Availability

The datasets used in this study are available, please track the link for access the data and materials used in this research: https://www.mediafire.com/folder/xyqqfbxl80xzg/Data.
